# Supplementary material for: Adequacy of Nutrient Intake and Malnutrition Risk in Older Adults: Findings from the Diet and Healthy Aging Cohort Study
Source: Nutrients. 2023 Aug 4;15(15):3446. doi: 10.3390/nu15153446 (PMC10421189; doi:10.3390/nu15153446)

Supplementary materials

Adequacy of nutrient intake and malnutrition risk in older adults: findings from the Diet and Healthy Aging cohort study

Kaisy Xinhong Ye[1,2], Lina Sun[3], Su Lin Lim[4], Jialiang Li[5], Brian K. Kennedy[2,6,7], Andrea Britta Maier[2,6,8], Lei Feng[1,2,*]

Table of Contents

***Text S1. Nutritional Knowledge questionnaire* 2**

***Figure S1. Flowchart of selection process.* 3**

Text S1. Nutritional Knowledge questionnaire

Nutrition knowledge

1. Diet can be related to certain diseases. Please answer the following questions based on what you know

(I) A diet high in fat is related to<br>(1) Gastritis<br>(2) Heart disease	True	False	Don't know
	1	2	N
	1	2	N
(II) A diet high in salt / sodium is related to<br>(3) Hypertension (high blood pressure) (4) Arthritis	True	False	Don't know
	1	2	N
	1	2	N
(III) A diet low in fiber is related to<br>(5) Heart disease (6) Constipation	True	False	Don't know
	1	2	N
	1	2	N
(IV) A diet low in calcium is related to<br>(7) Diabetes<br>(8) Osteoporosis	True	False	Don't know
	1	2	N
	1	2	N
(V) A diet low in iron is related to<br>(9) Anemia<br>(10) Heart disease diseases	True	False	Don't know
	1	2	N
	1	2	N
(VI) The consumption of preserved foods is related to (11) Rheumatism<br>(12) Some cancers	True	False	Don't know
	1	2	N
	1	2	N

2. Do you pay attention to dietary and nutrition information?
(1) Always (2) Sometimes (3) Seldom (4) I don't know

3. Your most important sources of nutrition information are:
   (1) Your children
   (2) Your spouse
   (3) Relatives, friends and neighbors
   (4) Book/newspaper/magazine, TV/radio, internet
   (5) Medical practitioners, nurses, dieticians
   (6) Courses or lectures
   (7) Salespeople

Figure S1. Flowchart of selection process.

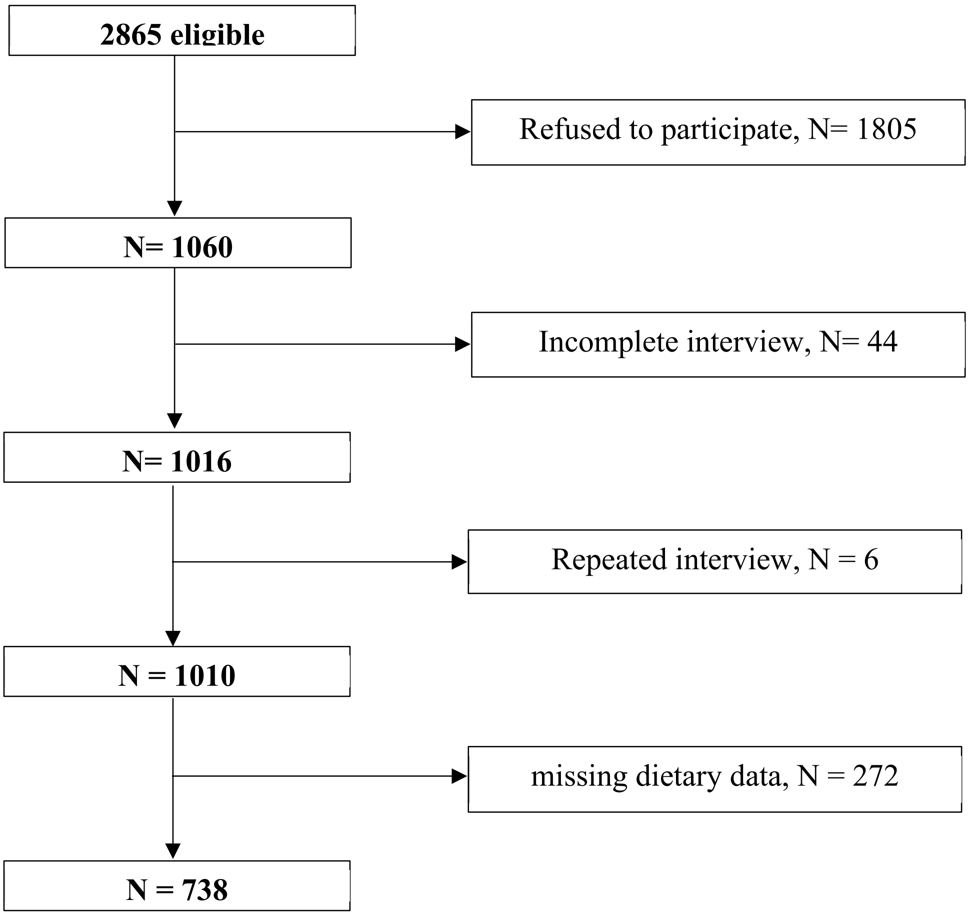

Supplement: Supplementary file 1 [file nutrients-15-03446-s001.zip › nutrients-2525223-supplementary.pdf]
